# Impact of the Arbuscular Mycorrhizal Fungus *Funneliformis mosseae* on the Physiological and Defence Responses of *Canna indica* to Copper Oxide Nanoparticles Stress

**DOI:** 10.3390/jof8050513

**Published:** 2022-05-16

**Authors:** Jie Luo, Qiuxia Yan, Guo Yang, Youbao Wang

**Affiliations:** 1 School of Ecology and Environment, Anhui Normal University, Wuhu 241000, China; lj26wlxy@163.com; 2 School of Yuanpei, Shaoxing University, Shaoxing 312000, China; yqx2014827615@126.com; 3 School of Life Science, Shaoxing University, Shaoxing 312000, China

**Keywords:** arbuscular mycorrhizal fungi, nano-CuO, antioxidant, stress-response gene

## Abstract

Copper oxide nanoparticles (nano-CuO) are recognized as an emerging pollutant. Arbuscular mycorrhizal fungi (AMF) can mitigate the adverse impacts of various pollutants on host plants. However, AMF’s mechanism for alleviating nano-CuO phytotoxicity remains unclear. The goal of this study was to evaluate how AMF inoculations affect the physiological features of *Canna indica* seedlings exposed to nano-CuO stress. Compared with the non-AMF inoculated treatment, AMF inoculations noticeably improved plant biomass, mycorrhizal colonization, leaf chlorophyll contents, and the photosynthetic parameters of *C. indica* under nano-CuO treatments. Moreover, AMF inoculation was able to significantly mitigate nano-CuO stress by enhancing antioxidant enzyme activities and decreasing ROS levels in the leaves and roots of *C. indica*, thus increasing the expression of genes involved in the antioxidant response. In addition, AMF inoculation reduced the level of Cu in seedlings and was associated with an increased expression of Cu transport genes and metallothionein genes. Furthermore, AMF inoculations increased the expression levels of organic acid metabolism-associated genes while facilitating organic acid secretion, thus reducing the accumulation of Cu. The data demonstrate that AMF–plant symbiosis is a feasible biocontrol approach to remediate nano-CuO pollution.

## 1. Introduction

Engineering nanomaterials (ENMs) are generally defined as ultra-fine particles with at least one dimension measuring less than 100 nm. They primarily consist of metals, metal oxides, and metalloids, which largely contain carbon and other elements. Nano-CuO is an indispensable engineering nanomaterial because of its prominent physical and chemical properties. Nano-CuO production has increased rapidly worldwide and has applications in the agricultural industries, next-generation medicine, plastics, textiles and paints, energies, fuel additives, the chemical and food industries, environmental remediation, electronics and optics, cosmetics, coatings, and consumer products [1]. With the increasing demand for nano-CuO, global production is estimated to be 1600 tons by 2025, of which approximately 25% will be released into the soil, air, and water systems [2]. Nanoparticle increase within groundwater and soil is likely to cause nanoparticles to accumulate within the tissues of plants. Moreover, nano-CuO has high toxicity to humans, plants, and other aquatic creatures (e.g., crustaceans, fish, algae, and mussels) [3,4,5]. 

Existing studies have reported the phytotoxicity of nano-CuO. For instance, nano-CuO significantly inhibited *Coriandrum sativum* seed germination [6]. Stampoulis et al. [7] found that nano-CuO stress reduced *Cucurbita pepo* biomass by 90%. In addition, nano-CuO stress reduced the length of *Brassica juncea*’s lateral and main roots [8]. According to Deng et al. [9], nano-CuO may damage and reduce the root cap, meristematic zone, and root tip development in *Allium cepa*. Nano-CuO exhibited stronger adsorption into the soil as compared with Cu ions. Nano-CuO may cause toxicity either by releasing Cu ions from particles or through direct interactions with plant tissues. As indicated by Shi et al. [10], nano-CuO can be absorbed and accumulate in plant cells. The accumulation of nano-CuO disrupts the integrity of cellular and subcellular organelles, ultimately changing the plant’s physiological processes. In addition, through nano-CuO accumulation, the metabolic activity and production of reactive oxygen species (ROS) in plant tissues can be impacted, thus damaging DNA [11]. When the concentration of nano-CuO is high, plant physiological, biochemical, and photosynthetic performance is impaired, resulting in cell disorder [10]. Metal nanoparticles can cause phytotoxicity by dissolving and releasing high concentrations of essential ions or producing excess ROS. However, the exact mechanisms by which plants defend against nano-CuO toxicity are not fully understood. 

Arbuscular mycorrhizal fungi (AMF) are obligate biotrophs that are widespread in the microbial population of the rhizosphere. They can establish sustained mutualist symbioses with 80% of vascular plant species and have a major impact on the structure and nutritional dynamics of plant roots [12]. AMF can help host plants survive by improving nutrient availability, notably phosphorus (P), increasing plant biomass [13]. Moreover, they also improve the physicochemical properties of soil and act as filters to block xenobiotics within their mycelium. As a result, AMF operate as physical barriers and sheaths for mycorrhizal plants, immobilizing heavy metals in plant roots. In addition to the generally recognized effects on metal immobilization within the cell wall of the intraradical and extraradical mycelium or fungal secreted compounds, including glomalin [14], it has been suggested that AMF re-regulate the expression of genes involved in plant metal transport processes [15]. Other studies also found that AMF can significantly alter the expression of P and arsenite transporters in plants [13]. For metal nanoparticles, previous studies found that AMF can reduce the phytotoxicity of Ag nanoparticles and zinc oxide nanoparticles by reducing metal bioavailability or the improvement of glomalin secretion, which reduces metal accumulation in plants [16,17]. However, it is still unclear how AMF influences plant gene expression to mediate the phytotoxicity of nanoparticles. Moreover, the mechanism governing AMF-mediated tolerance based on nano-CuO stress remains to be elucidated. 

*Canna indica* L. and AMF can create a stable symbiotic connection, with AMF colonization reaching up to 98% [18]. The use of AMF–*C. indica* symbiosis in the regeneration of polluted soil in mining zones demonstrated that AMF inoculation might substantially enhance heavy metal absorption from the contaminated soil [19]. This species is considered the most valuable plant for metal phytoremediation technology in urbanized areas. However, there is no research on the use of AMF*–C. indica* symbiosis to reduce copper oxide nanoparticle toxicity. Thus, the present study aimed to investigate the roles of AMF in influencing the responses of *C. indica* to different concentrations of nano-CuO. The biomass, photosynthetic parameters, antioxidant enzyme activity, and organic acids of *C. indica* seedlings were measured. Genes potentially involved in the antioxidant response, Cu transport, metallothionein, and organic acid metabolism of *C. indica* roots were studied. We hypothesized that: (1) AMF facilitate *C. indica* seedling establishment under nano-CuO stress; (2) AMF induce a high-efficiency ROS scavenging mechanism that protects *C. indica* seedlings exposed to nano-CuO stress from oxidative damage; (3) AMF facilitate organic acid generation and secretion to chelate nano-CuO. Our findings provide a theoretical basis that furthers our understanding of AMF regarding improving the phytoremediation of ENM-contaminated soils.

## 2. Materials and Methods

### 2.1. Preparation of Plant, Soil, and Fungal Materials

**Figure 1:**
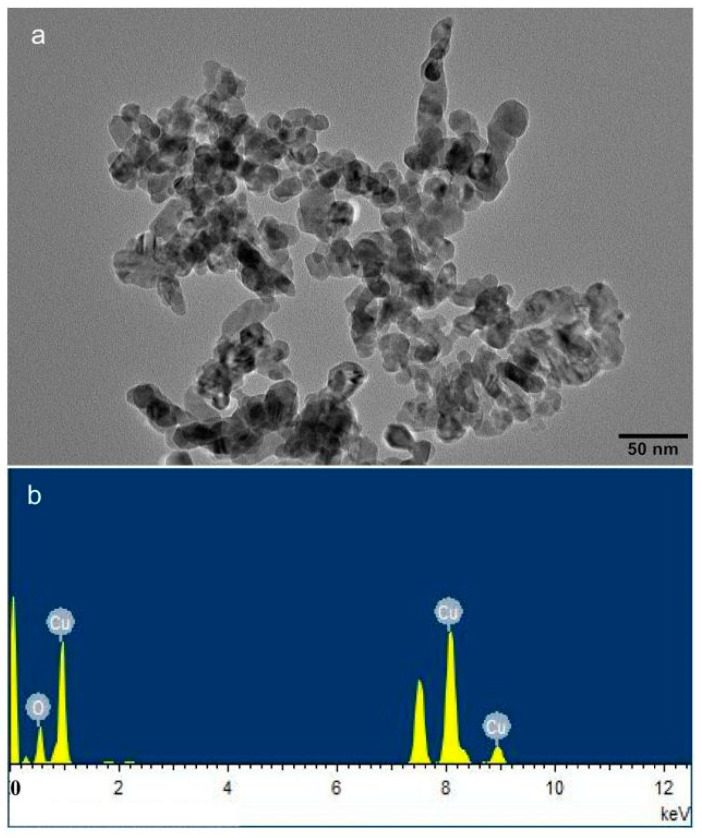
Characterization of nano-CuO: (**a**) TEM micrograph of nano-CuO; (**b**) spectrogram of the selected area.

Annual tubers of *C. indica* L. were purchased at Xiaoshan flower and tree market in Zhejiang Province, China. The Institute of Root Biology of Changjiang University provided *Funneliformis mosseae.* The AMF inoculum received a 5-month spread on *Trifolium repens*, grown in high-temperature sterilized sandy soil. Meanwhile, the non-AMF inoculum was prepared with the same sterilized substrate, and host plants were cultured under the same conditions. The prepared AMF inoculum, including extraradical hyphae and cultivation substrates containing spores (nearly 200 g^−1^), was air-dried. The planting soil (mainly humus), which was purchased in the market, was passed through a 4 mm mesh sieve and, before use, underwent 2 h of sterilization at a temperature of 121 °C. The soil properties were as follows: pH 6.86, organic matter 25.0%, and the available N, P, and K were 166.20, 50.34, and 213.74 mg·kg^−1^, respectively. Nano-CuO (copper II oxide) was purchased from Sigma–Aldrich, USA. In this paper, we used transmission electron microscopy (TEM) (JEM-2100F, Joint-stock Company, Japan) to characterize the nano-CuO properties and the particle size distribution. We carried out elemental analysis using energy-dispersive X-ray spectroscopy (TEM–EDX) using an energy-dispersive spectrometer EDAX (XFlash 5030T). The electron microscope observation showed that the sample was composed of spherical particles with an average diameter of less than 50 nm (Figure 1).

### 2.2. Growth Conditions and Experimental Design

In late February 2021, the surfaces of the tubers were sterilized with 75% (*v*/*v*) ethanol for 30 s, rinsed several times with sterile distilled water, and then planted into polystyrene pots (8 × 8 × 16 cm) with 1 kg of a sterile substrate. In addition, half of the pots were inoculated with AMF inoculum and the other half with non-AMF inoculum, using 20 g of inoculum in each pot. In the culture media, AMF and non-AMF inoculum were uniformly distributed. The experimental pots were placed in a greenhouse at an average temperature of 25 ± 2 °C (day/night), with a relative humidity of 60−80%. The photon flux density was in the range 1200 ± 200 mol m^−2^ s^−2^ with a 14/10 h (light/night) photoperiod.

A two-factorial design with nano-CuO and AMF inoculation as factors was performed. Three levels of nano-CuO (0, 300, and 600 mg kg^−1^ of sterilized soil) were added, and all the above were either AMF inoculated (+AMF) or non-AM fungal inocula (−AMF). Each treatment was repeated 10 times. After 60 days of *C. indica* planting, nano-CuO were dispersed in water with a sonicator (600 W, 20 min) and then dripped onto the soil surface to ensure that the particles were fully mixed into the soil.

The pots were irrigated weekly with 20 mL of half-strength Hoagland’s nutrient solution. All *C. indica* plants were harvested after 100 days of growth. We found a remarkable difference in terms of plant growth between treatments. Root and leaf samples were stored at 4 °C or −80 °C for the physiological index determination and gene expression analysis, respectively.

### 2.3. Leaf Chlorophyll Content and Photosynthetic Parameters

According to the method of Porra et al. [20], the top leaves were selected for the chlorophyll content measurement (chlorophyll a, b, and total chlorophyll). With the use of the fluctuating natural outdoor light, the measurements of transpiration rate (T_r_), stomatal conductance (G_s_), intercellular CO_2_ concentration (C_i_), and net photosynthetic rate (P_n_) were performed from 9:30 am to 10:30 am. The photosynthetic parameters were monitored in LI-6400 (Li-COR, Lincoln, NE, USA) at 10 s intervals.

### 2.4. Morphological Parameters

*C. indica* plants were harvested on the 100th day of the experiment, each plant was rinsed with deionized water, and the stem and root length were determined. Thereafter, the samples were divided into aboveground parts (shoot) and underground parts (root). The biomass was weighed after being oven-dried at 60 °C for 48 h.

### 2.5. AM Colonization Rate

Roots were cut into 1 cm pieces. Then, the cut roots were made transparent with 10% KOH in a 90 °C water bath for 20 min. Subsequently, the sample underwent 5 min acidification treatment with 2% HCl and then stained using 0.01% fuchsin acid [21]. The mycorrhizal colonization rate was determined using the gridline intercept approach, according to the method of Giovannetti and Mosse [22].

### 2.6. Cu and P Content Analysis

We ground the dried plant tissue samples into powders and passed them through a 60 mesh sieve. A total of 0.2 g of the sample was subsequently digested with an acid mixture (HNO_3_/HClO_4_ = 3:1). The Cu concentration of the plant sample was analyzed using ICP-OES (Agilent 5800). The total P content was evaluated using molybdenum-blue colorimetry after the sample was digested with a combination of H_2_SO_4_ and H_2_O_2_ [23].

### 2.7. Antioxidant Enzyme, Lipid Peroxidation (MDA), and Reactive Oxygen Species (ROS)

Fresh leaf and root tissues (0.4–0.5 g) were homogenized in a buffer solution (50 mM potassium phosphate, pH 7.8) under pre-chilled conditions to assess the antioxidant enzyme activity. The homogenate was centrifuged at 10,000× *g* for 20 min at 4 °C, and the supernatant was utilized for further investigation. According to Li et al. [24], the overall superoxide dismutase (SOD, EC 1.15.1.1) activity was determined by measuring the enzyme’s capability to inhibit the photochemical reduction of nitroblue tetrazolium. The peroxidase (POD, EC 1.11.1.7) activity was determined based on guaiacol oxidation [25], and the catalase (CAT, EC 1.11.1.6) activity was measured following the degradation of H_2_O_2_ for 60 s at 240 nm [26]. The glutathione reductase (GR, EC 1.6.4.2) activity was estimated by Jiang and Zhang [27]. Malonaldehyde (MDA) content was determined by Naeem et al. [28]. Hydrogen peroxide (H_2_O_2_) contents were measured at 390 nm, and a standard curve was used to calculate the H_2_O_2_ level according to Velikova et al. [29]. Superoxide radicals (O_2_^−^) were determined according to Jiang and Zhang’s [27] method. SOD, POD, CAT, GR, MDA, O_2_^−^, and H_2_O_2_ analyses were conducted in three biological replicates.

### 2.8. Collection and Measurement of Organic Acids Released from Roots

The approach of Ding et al. [30] and Yang et al. [31] was used to enhance the extraction and determination of root exudates. After washing the dirt from the plant roots with tap water, the roots were rinsed 3–4 times with distilled water until they were clean. The roots were then steeped in a thymol solution with a concentration of 5 mg L^−1^ for 5 min. After that, the roots were transferred into a collecting device containing 500 mL of distilled water and coated in tin foil to protect them from light. Root exudates were gathered over 6 h of natural light ventilation. After the root system was removed, the collected solution was filtered through a 0.45 m filter to remove the abscission from the root system. Rotation at 50 °C evaporated the collected solution, which was then concentrated to 10 mL. The concentrated solution was filtered through a 0.45 m membrane and cryo-preserved at −20 °C for later use. The concentration of organic acids (oxalic acid, malic acid, and citric acid) was measured using HPLC with an ion-exclusion column (Shodex Rspak Kc811, 300 mm × 8 mm) equipped with a guard column (50 mm × 8 mm) at a wavelength of 210 nm.

### 2.9. Gene Expression Analysis

We performed the total RNA extraction using 0.1 g of fresh roots and leaves from *C. indica* seedlings under different treatments with the plant RNA extraction tool (Sangon Biotech, Shanghai, China) in strict accordance with the manufacturer’s instructions. cDNA was synthesized with the PrimerScript^TM^ RT reagent Kit (Takara, Kyoto, Japan). The real-time quantitative PCR was conducted using three biological and two technical replicates with the SYBR PremixEx Taq (Takara) in a Bio-Rad CFX96 real-time PCR system (Bio-Rad, Hercules, CA, United States). The conditions of the qRT-PCR included 30 s pre-denaturation at a temperature of 95 °C, followed by a 5 s 40 cycle denaturation at a temperature of 95 °C and a 30 s annealing and extension process at a temperature of 58 °C, in accordance with Li et al. [32]. Premier 5.0 was used to design the primers for RT-PCR amplification (Table S1). The 18S rRNA gene was selected as an internal control gene to perform relative qRT-PCR. We obtained the relative expression level using the 2^−ΔΔCt^ method, according to Livak and Schmittgen [33].

### 2.10. Statistical Analysis

Plots were generated in excel and R language with the ggplot2 package. We carried out significant experiments using the Tukey–Kramer test using the SPSS 20.0 software with a significance level of 0.05.

## 3. Results

### 3.1. Plant Growth, AM Colonization, and Cu Content of Seedlings

**Figure 2:**
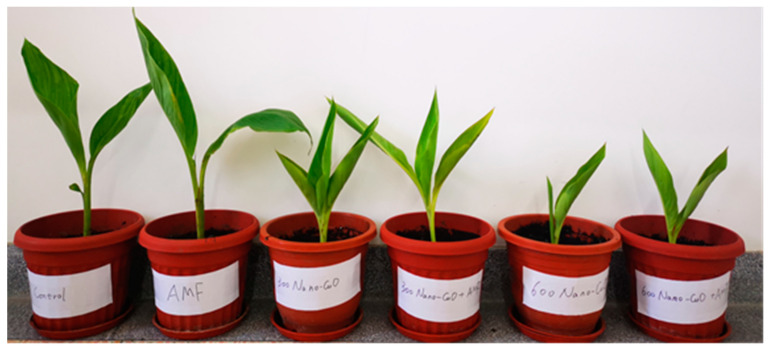
AMF enhanced *C. indica* seedling growth under nano-CuO stress. The test treatments from left to right: control treatment (without Cu or AMF); AMF (without Cu); nano-CuO (300 mg kg^−1^); AMF + nano-CuO (300 mg kg^−1^); nano-CuO (600 mg kg^−1^); and AMF + Nano-CuO (600 mg kg^−1^).

With the elevation of nano-CuO levels within the soil, the *C. indica* root and shoot dry biomass and length exhibited remarkable reductions (Figure 2, Table 1). In the absence of nano-CuO in the soil media, AMF-inoculated plants exhibited higher biomass and length values than non-AMF inoculated plants, including root length, shoot dry mass, root dry mass, and shoot length. Under 300 mg kg^−1^ nano-CuO stress, when seedlings were treated with AMF, the total dry mass, root dry mass, shoot dry mass, shoot length, and root length were all significantly increased by 12.3%, 23.3%, 14.6%, 11.9%, and 10.1%, respectively, in contrast with the non-AMF treatment plants (Table 1). In contrast, AMF-inoculated plants exposed to 600 mg kg^−1^ nano-CuO demonstrated no difference in terms of biomass and length relative to non-AMF inoculated plants. As revealed by the above result, based on normal and nano-CuO conditions, AMF facilitated *C. indica* seedling growth.

**Figure 3:**
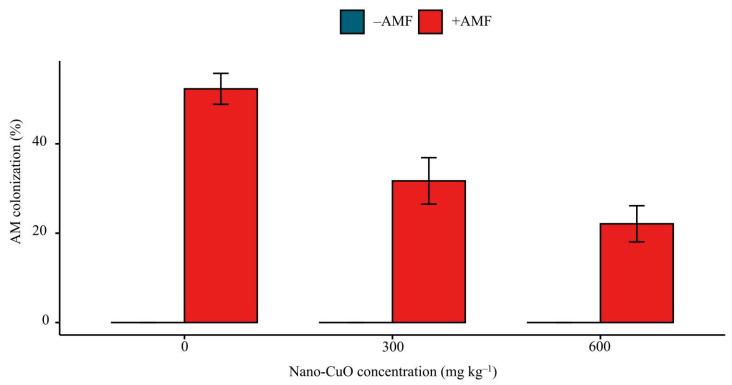
AM colonization of *C. indica* roots under nano-CuO stress.

We did not find AM colonization within non-inoculated seedling roots of *C. indica*, while *C. indica* roots were colonized by *F. mosseae* in the inoculated seedlings. All AMF inoculation treatments showed mycorrhizal colonization in *C. indica* roots. The mycorrhizal colonization varied among different nano-CuO treatments, ranging from 22.1% to 52.5% (Figure 3). The highest colonization was found in the AM fungal-inoculated treatment plants without nano-CuO stress. The nano-CuO concentration in the soil had a negative impact on AM colonization.

Increased nano-CuO levels in the substrate increased Cu content while decreasing P concentration in *C. indica* shoots and roots. Under nano-CuO stress and AM fungal inoculation circumstances, the Cu content in shoots and P content in roots of *C. indica* plants did not differ from the non-AMF inoculation treated plants. However, the Cu content in the *C. indica* roots decreased by 20.1% and 11.7% under 300 and 600 mg kg^−1^ nano-CuO stresses, respectively (Table 1). The P content in the shoots of *C. indica* inoculated with AMF was significantly higher than that in the treated plants without AMF under 0, and 300 mg kg^−1^ nano-CuO stresses, but AMF inoculation had no influence on the P content in the *C. indica* roots under the 600 mg kg^−1^ nano-CuO stress.

### 3.2. Leaf Chlorophyll Contents and Photosynthetic Parameters

The chlorophyll content of leaf samples from each treatment was determined. According to Table 2, with the elevation of nano-CuO levels in the growth media, leaf chlorophyll contents decreased. AMF inoculation significantly upregulated leaf chlorophyll contents under the medium nano-CuO level (300 mg kg^−1^), and chlorophyll a, chlorophyll b, and total chlorophyll contents were elevated by 17.65%, 28.13%, and 20.00%, respectively, as compared to the non-AMF inoculation plants. However, AMF inoculation insignificantly impacted leaf chlorophyll contents under the high nano-CuO concentration (600 mg kg^−1^) and without nano-CuO stress.

The P_n_, G_s_, C_i_, and T_r_ were significantly influenced by the nano-CuO treatment (Table 2). The P_n_, G_s_, and T_r_ decreased as nano-CuO in the soil increased. C_i_ exhibited the opposite trend. When there was no nano-CuO stress, AMF inoculation insignificantly impacted plant photosynthetic parameters in relation to the non-AMF inoculation treatment plants. Nevertheless, under the 300 mg kg^−1^ or 600 mg kg^−1^ nano-CuO stress in the soil, AMF inoculation significantly improved the P_n_, G_s_, and T_r_ in the *C. indica* leaves.

### 3.3. Antioxidant Enzyme Activities, Lipid Peroxidation (MDA), and Reactive Oxygen Species (ROS)

**Figure 4:**
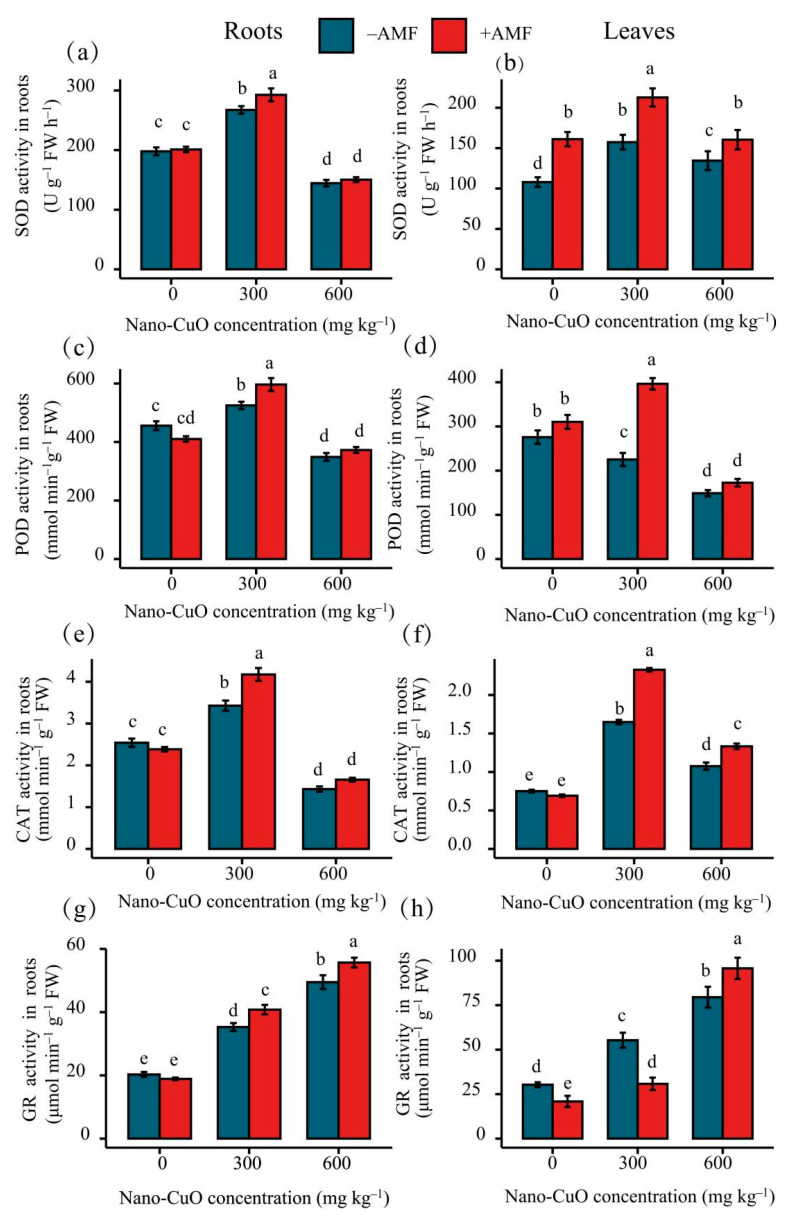
Effects of AMF on the antioxidant enzymes of *C. indica* seedlings under nano-CuO stress. Total superoxide dismutase (SOD) activity in *C. indica* roots (**a**) and leaves (**b**); peroxidase (POD) activity in *C. indica* roots (**c**) and leaves (**d**); catalase (CAT) activity in *C. indica* roots (**e**) and leaves (**f**); glutathione reductase (GR) activity in *C. indica* roots (**g**) and leaves (**h**). Different lowercase letters show significant differences between treatments (*p* < 0.05).

Nano-CuO stress at 300 mg kg^−1^ increased SOD, POD, and CAT activities in the leaves and roots of *C. indica* plants. Subsequently, we found a significant decrease in the activity of the above three antioxidant enzymes at the highest level of nano-CuO (Figure 4). Antioxidant enzyme activities, including GR, significantly increased with the increase in nano-CuO levels. In substrates with no nano-CuO stress, AMF inoculation treatment insignificantly impacted GR, CAT, POD, and SOD activities. For the medium nano-CuO level (300 mg kg^−1^), AMF inoculation significantly increased the activities of SOD, POD, CAT, and GR. The activities of SOD, POD, CAT, and GR were increased by 9.46%, 13.57%, 21.76%, and 15.58%, respectively, as compared to the non-AMF inoculation plants. However, the AMF inoculation treatment had no significant influence on the activities of SOD, POD, or CAT, although it affected GR activity under a high level of nano-CuO treatment (600 mg kg^−1^).

**Figure 5:**
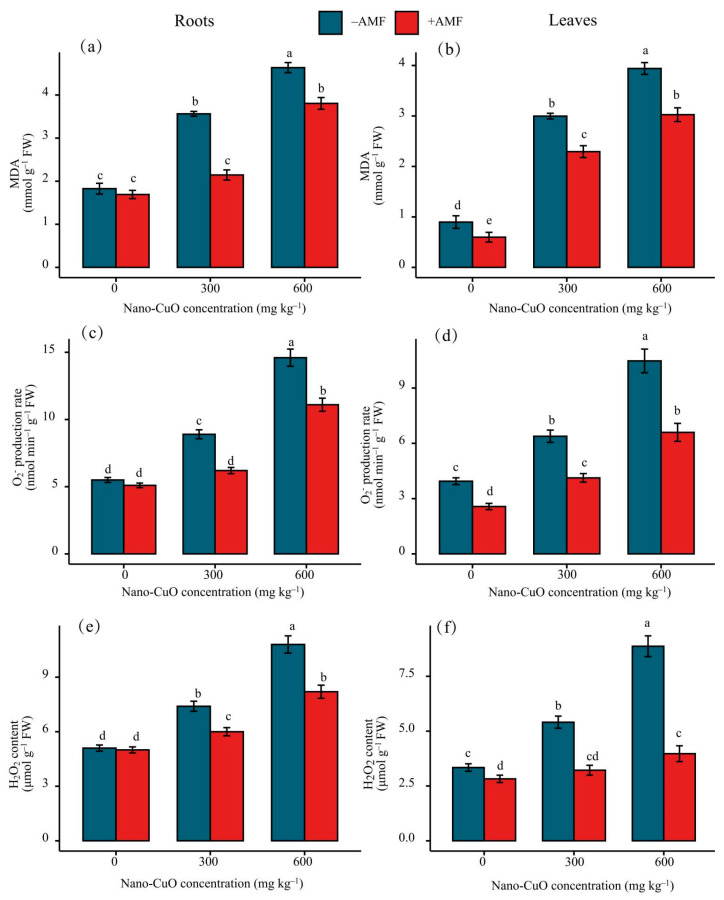
Effects of AMF on MDA and ROS in leaves of *C. indica* under nano-Cu stress. Malonaldehyde (MDA) content in *C. indica* roots (**a**) and leaves (**b**); hydrogen peroxide (H_2_O_2_) contents in *C. indica* roots (**c**) and leaves (**d**); superoxide radicals (O_2_^−^) in *C. indica* roots (**e**) and leaves (**f**). Different lowercase letters show significant differences between treatments (*p* < 0.05).

The plants exposed to nano-CuO had significantly elevated levels of ROS (H_2_O_2_ and O_2_^−^) in the root and leaf tissues compared to the control plants (Figure 5). When the AMF treatment was performed, H_2_O_2_ and O_2_^−^ accumulation showed a remarkable reduction compared to the non-AMF inoculated treatment plants. MDA, an indicator of lipid peroxidation, was examined to verify whether excessive nano-CuO caused oxidative stress in the leaves and roots of *C. indica* seedlings (Figure 5). Under the nano-CuO stress, the MDA content improved with the increase in nano-CuO levels in the growth media, which demonstrated that the permeability of the cell membrane and the conductivity of outer permeation increased. However, the MDA contents in the leaves and roots of the seedlings after AMF inoculations decreased significantly compared to the control group. In this study, AMF was shown to alleviate the level of lipid peroxidation induced by nano-CuO stress.

### 3.4. Impacts on the Organic Acid Content in Roots Arising from Stress

**Figure 6:**
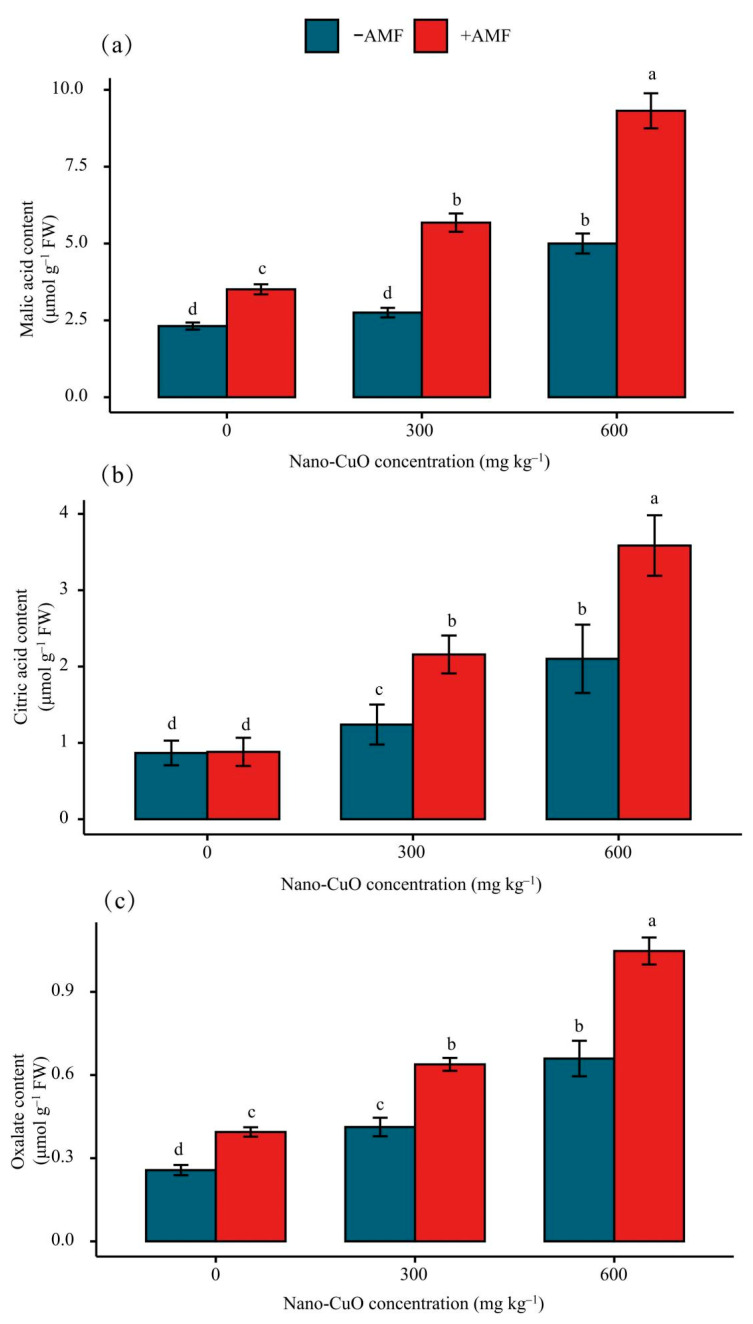
The function of AMF in the secretion of citric acid (**a**), malic acid (**b**), and oxalate (**c**) in *C. indica* roots in response to nano-CuO stress. Different lowercase letters show significant differences between treatments (*p* < 0.05).

A plant root’s organic acid secretion significantly affects a plant’s responses to Cu toxicity. We measured the concentrations of malic, citric, and oxalic acids in *C. indica* roots. The results revealed remarkable elevations of the concentrations of malic, citric, and oxalic acids in *C. indica* roots in response to nano-CuO stress (Figure 6). Moreover, the concentrations of malic, citric acids, and oxalic acid in the AMF treatment plants were noticeably higher than those in the nano-CuO treatment plants (Figure 6).

### 3.5. Impacts on Gene Expression Arising from AMF in Response to Nano-CuO Stresses

**Figure 7:**
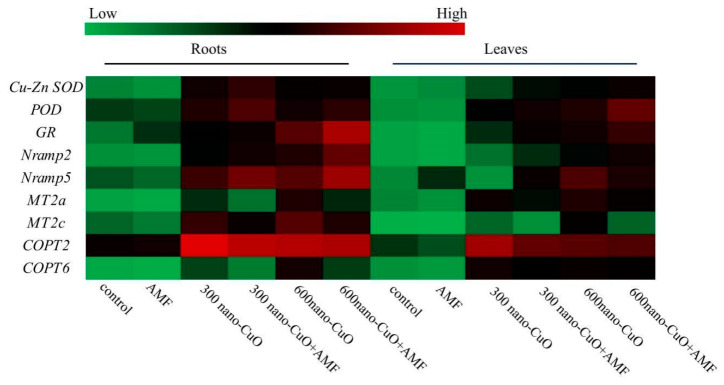
The effect of AMF on the expression of antioxidant enzyme-related genes and metal ion transport-related genes under nano-CuO stress in *C. indica* roots and leaves. Cu-Zn SOD: superoxide dismutase (Cu-Zn); GR: probable glutathione S-transferase parA; POD: peroxidase; Nramp: metal transporter Nramp; MT: metallothionein-like protein; COPT: copper transporter. Different colors represent gene expression levels from lowest (green) to highest (red) in the entire database. The RT-PCR assays were performed in three independent biological replicates and three technical replicates.

To explore the molecular regulation of *C. indica* seedlings in the AMF inoculation treatment plant group based on nano-CuO stress, the expressions of genes that play a role in metal transport (*Nramp2*, *Nramp5*, *MT2a, MT2c, COPT2,* and *COPT6*) and the antioxidant response (*Cu-Zn SOD*, *POD*, and *GR*) were assessed. Figure 7 illustrates the relative expression levels of genes in *C. indica* roots due to different stresses. As revealed by the qRT-PCR analysis, *C. indica* leaves and roots exhibited elevated relative expression levels of *Nramp2*, *Nramp5*, *MT2a*, *MT2c, Cu-Zn SOD*, *POD*, and *GR* resulting from nano-CuO stress, as compared with the control. Without nano-CuO in the media, AMF inoculation treatment insignificantly impacted the relative expression levels of *Nramp2*, *Nramp5, MT2a, MT2c*, *Cu-Zn SOD*, *POD*, and *GR*. Nano-CuO at 300 mg kg^−1^ and AMF inoculation significantly improved the relative expression levels of *Nramp2*, *Nramp5*, *Cu-Zn SOD*, *POD,* and *GR*, but not *MT*, as compared with the non-inoculated AMF treatment plants. Inoculation with AMF significantly increased the antioxidant response gene (*Cu-Zn SOD* and *GR*) and *Nramp* expressions, but not *MT* expression, under a high level of nano-CuO (600 mg kg^−1^).

**Figure 8:**
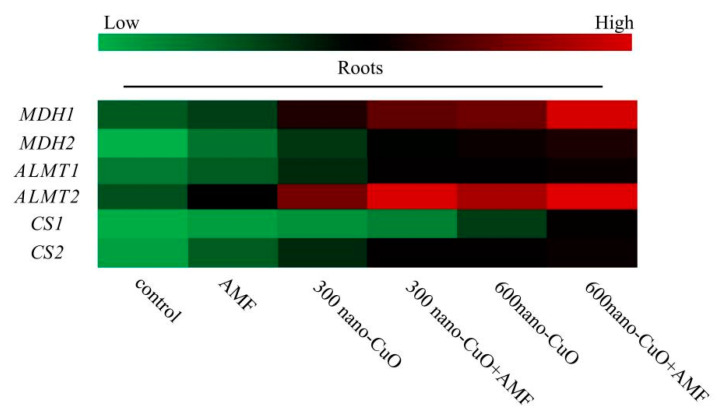
The effect of AMF on the expression of malic acid and citric acid metabolism-related genes under nano-CuO stress in *C. indica* roots. MDH: malate dehydrogenase; ALMT: aluminum-activated malate transporter; CS: citrate synthase. Different colors represent gene expression levels from lowest (green) to highest (red) in the entire database. The RT-PCR assays were performed in three independent biological replicates and three technical replicates.

AMF noticeably elevated the transcription levels of organic acid synthase-associated genes in *C. indica* seedlings, consistent with the results of the organic acid concentration analysis. The expressions of *MDH* and *CS* in the AMF treatment plants were significantly higher than those in the non-AMF treatment plants (Figure 8). Thus, it was proposed that AMF application might promote the secretion of malic and citric acids in *C. indica* roots in response to nano-CuO stress. *ALMT* (aluminum-activated malate transporter-related genes) are key genes in malate secretion in the higher plant. The expression levels of *AMLT1* and *AMTL2* were remarkably upregulated in *C. indica* seedling roots exposed to nano-CuO stress, while exogenous AMF further increased AMLT expression within *C. indica* seedling roots.

## 4. Discussion

### 4.1. AMF Significantly Facilitated C. indica Seedling Establishment in Response to Nano-CuO Stress

AMF easily form symbiotic relationships with different types of plants and are capable of noticeably boosting the development of plants through an improvement in the density of extraradical mycelium beyond the nutrient depletion zone around the root, thus facilitating the absorption of minerals and water [34]. According to current findings, *C. indica* and AMF (*F. mosseae*) can produce a steady symbiotic relationship [18,35,36]. In this paper, we found considerable vesicles and hyphae within the root samples. AMF colonization of *C. indica* roots reached 50% in spring, much higher than the colonization rate recorded in other studies [36]. The *Funneliformis* genus is capable of more significantly resisting exogenous stresses and quickly colonizing the plant root [37]. It has been reported that *F. caledonium* and *F. versiforme* can alleviate the adverse impacts arising from heavy metals [17]. Moreover, several studies report positive and negative impacts arising from ENMs on AM colonization [38,39]. The findings in this paper support the perspective that metal oxide nanomaterials decrease AM colonization [16], possibly because nano-CuO inhibits the propagation and diffusion of fungal spores. However, nano-CuO’s impacts on AM colonization seem to depend on the nano-CuO concentration. High nano-CuO (600 mg kg^−1^) stress significantly inhibited the AMF colonization rate in the study.

AMF are capable of alleviating the damage attributed to ENM stress and promoting host plants to be more resistant and grow more rapidly [40]. This is in accordance with our findings which revealed the impacts arising from AMF treatment on photosynthesis, plant growth, and chlorophyll content in *C. indica* seedlings under nano-CuO stress. Under Fe_3_O_4_NPs stresses, the levels of certain nutrient elements (e.g., Ca, K, P, and N) within the aerial part of maize seedlings subjected to AMF inoculation were elevated by over 10% [41]. This arose from the sizeable extra root mycelial system that emerged after AMF symbiosis and the formation of the anise root system. The plant root’s acquisition range of nutrients is broadened due to extra mycorrhizal hyphae growth. As a result of AMF inoculation, nutrient absorption throughout the roots and the cycling process of rhizosphere nutrients are enhanced, which can lead to an improvement of the plant’s nutrition status [42].

*C. indica* is invasive in numerous countries and potentially invasive in other countries/regions where it is cultivated. AMF play a significant role in the successful invasion of alien plant species because of their capacity to stimulate growth [43]. Qi et al. [44] discovered that AMF improved *Solidago canadensis*’ ability to absorb phosphorus under stress circumstances, allowing them to shift their resource allocation strategy to favor more aboveground biomass. This was comparable to our findings, which showed that AMF inoculation can increase aboveground biomass and P content of *C. indica* under nano-CuO stress. The findings also show that AMF improves invasive species’ competitiveness by increasing plant P accumulation.

### 4.2. AMF Led to a High-Efficiency ROS Scavenging Mechanism for the Protection of the C. indica Seedlings Exposed to Nano-CuO Stress from Oxidative Damage

Plants can become more tolerant to heavy metal stress through various biochemical channels (e.g., particular proteins managing water flux, ions, free radicals, and osmotically dynamic metabolites) [45]. As indicated by the results in this paper, plants inoculated with AMF exhibited an increased concentration of MDA under nano-CuO conditions. In this paper, the accumulation of O_2_^−^ and H_2_O_2_ due to nano-CuO stress decreased after AMF inoculation of *C. indica* seedling. ROS scavenging is important to reduce oxidative stress and maintain normal plant metabolism [46,47]. Enzymatic antioxidants (e.g., GR, CAT, POD, and SOD) can significantly alleviate excessive ROS and balance generation and scavenging [48,49]. AMF-inoculated plants exhibited upregulated *GR*, *POD,* and *Cu-Zn SOD* expressions and improved antioxidant enzyme activity under nano-CuO stress. Thus, it was demonstrated that AMF led to a high-efficiency ROS elimination mechanism to protect the *C. indica* seedlings under wide-ranging oxidative damage. AMF inoculation led to a huge improvement in enzyme activity and the expressions of genes that play a role in ROS homeostasis due to abiotic stresses; thus, the host plants could more significantly protect themselves against oxidative stresses. There was a correlation between the above responses and plant tolerance to abiotic stress [50]. Furthermore, non-enzymatic antioxidants are also the key factor that provide tolerance against heavy metal stress [51]. Ascorbate and glutathione are vital in reducing Pb(II) ions toxicity in *Scenedesmus dimorphus* [52]. The role of non-enzymatic antioxidants in nano-CuO stress will be investigated in future studies.

### 4.3. AMF Inhibited Cu Uptake in C. indica Seedlings to Alleviate Nano-CuO Stress

The mitigation of metal-induced phytotoxicity by AMF is often significantly correlated with metal acquisition inhibition by plants or the prevention of metal transport from plant roots to shoots [53]. A recent study showed that under ZnONP treatment, AMF-inoculated plants increased biomass and decreased Zn accumulation as compared to non-AMF-inoculated plants [16]. We reported a remarkable reduction in Cu acquisition in the roots and shoots of mycorrhizal plants under nano-CuO treatment, demonstrating that AMF likely protects the plant by reducing their Cu acquisition rather than preventing Cu transportation to the plant shoots. The AMF hyphae combined with Cu in the soil increased the secretion of glomalin, produced glomalin-associated proteins, and decreased the uptake of Cu by the plant roots [54,55].

AMF may regulate the expression of genes that play a role in the metal transportation of their hosts [56]. It was considered that metals are actively carried by the proteins responsible for metal transport from the soil to the roots [57]. Existing studies reported that Cu or nano-CuO is likely absorbed through the membrane channels (e.g., aquaporins) [58]. According to the results in this paper, the expressions of genes involved in metal transport (*Nramp*, *COPT*) in *C. indica* roots were downregulated in AMF-inoculated plants compared with non-AMF-inoculated plants, which is in accordance with a recent study [59]. Similarly, it was reported that a gene encoding metallothionein (*MT*) decreased within the roots of AMF inoculation-treatment plants. Extensive studies demonstrated that *MT* can adjust metal homeostasis and create a vital cellular mechanism related to metal detoxification [13,60]. According to Ouziad et al. [13], AMF led to decreased MT gene expression due to the reduction in metal concentrations in mycorrhizal plants.

### 4.4. AMF Facilitated Organic Acid Generation and Secretion to Chelate Nano-CuO

Root secretion of organic acid (OA) anions (e.g., citric acid, malic acid, and oxalic acid) is significantly correlated with the plant’s response to heavy metal stress [61]. However, organic acids from the roots exhibit differences according to the species, including aluminum-induced citric acid secretion in maize [62] and rice beans [63], malate secretion in wheat [64], and oxalate secretion in buckwheat [65]. In contrast, triticale and oilseed rape can secrete malate and citrate simultaneously [61]. In this paper, nano-CuO stress-induced malate, citrate, and oxalate secretion in *C. indica* roots, while AMF treatment mainly increased the secretion of malate and citrate under nano-CuO stress conditions. Moreover, AMF treatment noticeably led to a corresponding gene expression (*MDH*, *CS*) in *C. indica* roots under nano-CuO stress. AMF inoculation can increase OA anion secretion by improving the malate and citrate synthesis ability, thus enhancing the nano-CuO stress tolerance. Rice bean roots consisted of two phases of Al-induced citrate secretion [66]. In fact, citrate and malate secretion patterns within *C. indica* roots under the regulation of AMF should be studied in depth. Transporters or anion channels play an important role in the secretion of OA. OA transporters within the higher plant can fall into MATE and ALMT, thus separately mediating citrate and malate secretion [67]. *GmAMLT1*, *AtALMT1,* and *TaALMT1* are the genes that are reported to be central to malate secretion in soybean, Arabidopsis thaliana, and wheat [57,68]. ALMT’s physiological function correlates with mineral uptake, seed development, ion homeostasis, and heavy metal resistance [69]. We provide evidence that the expression levels of *AMLT1* and *AMTL2* were remarkably upregulated in *C. indica* seedling roots exposed to nano-CuO stress, while exogenous AMF further increased AMLT expression within *C. indica* seedling roots. The data confirmed that AMF inoculation treatment regulates citrate and malate secretion within anionic channels; therefore, an unknown signaling channel is likely involved.

## 5. Conclusions

Under nano-CuO treatments, AMF symbiosis dramatically increased plant biomass, P content, leaf chlorophyll contents, photosynthetic parameters, and antioxidant enzyme activities of *C. indica*, in parallel with elevated expressions of antioxidant response genes (*Cu-Zn SOD*, *POD*, *GR*). Furthermore, AMF may effectively alleviate nano-CuO stress by lowering MDA, H_2_O_2_, and O_2_^−^ levels in *C. indica* leaves and roots. Moreover, AMF inoculation decreased the Cu concentration in plant leaves and roots while also lowering the expression of genes involved in Cu transport (*COPT*) and metallothionein (*Nramp*, *MT*). In addition, AMF inoculations increased the production of organic acids (malic, citric, and oxalic acids) and upregulated the expression of genes involved in organic acid metabolism (*MDH*, *ALMT*, and *CS*), lowering Cu buildup. These findings reveal that AMF application improved the growth capacity and alleviated nano-CuO-induced phytotoxicity in *C. indica* seedlings by inducing antioxidant enzyme activities and organic acid secretion and suppressing excessive ROS generation. This research proposes a novel approach to addressing ENMs contamination in soil.

## Figures and Tables

**Table 1:** Effects of AMF on the biomass, the Cu content, and the P content in tissues of *C. indica* seedlings under nano-CuO stress.

Nano-CuO	Treatment	Shoot dry	Root Dry	Shoot Height	Root Length	Cu Content in Shoots	Cu Content in Roots	P Content in Shoots	P Content in Roots
Levels mg kg^−1^		Mass (g)	mass (g)	(cm)	(cm)	(mg kg^−1^, DW)	(mg kg^−1^, DW)	(mg g^−1^, DW)	(mg g^−1^, DW)
0	−AMF	2.63 ± 0.19b	0.65 ± 0.09b	36.9 ± 1.8a	22.3 ± 1.3a	8.45 ± 0.21c	33.52 ± 0.3e	2.41 ± 0.08bc	2.06 ± 0.04a
	+AMF	3.13 ± 0.21a	0.79 ± 0.06a	38.6 ± 1.9a	24.5 ± 1.5a	8.19 ± 0.11c	33.92 ± 0.056e	2.79 ± 0.05a	2.11 ± 0.04a
300	−AMF	2.06 ± 0.13d	0.46 ± 0.07c	27.5 ± 1.4c	15.1 ± 0.9c	68.37 ± 3.11b	218.73 ± 0.34c	2.32 ± 0.07c	1.94 ± 0.07ab
	+AMF	2.35 ± 0.08c	0.60 ± 0.06b	31.2 ± 1.6b	16.8 ± 1.0b	57.16 ± 4.62b	174.33 ± 1.72d	2.55 ± 0.08b	2.01 ± 0.06a
600	−AMF	1.59 ± 0.05e	0.31 ± 0.04d	22.6 ± 1.1d	10.9 ± 0.6d	88.05 ± 2.71a	278.3 ± 3.31a	2.02 ± 0.04d	1.74 ± 0.05c
	+AMF	1.71 ± 0.04e	0.39 ± 0.02d	24.5 ± 1.2cd	12.3 ± 0.7d	80.61 ± 2.52a	245.71 ± 5.01b	2.11 ± 0.05d	1.80 ± 0.07bc

Data are the means of three replicates (mean ± SE). Different lowercase letters show significant differences between treatments at *p* < 0.05.

**Table 2:** Effects of AMF on the photosynthetic parameters and chlorophyll contents of *C. indica* seedlings under nano-CuO stress.

Nano-CuO Content	Treatment	P_n_	G_S_	C_i_	T_r_	Chlorophyll a	Chlorophyll b	Total Chlorophyll
mg kg^−1^		μmol m^−2^ s^−1^	μmol m^−2^ s^−1^	mmol m^−2^ s^−1^	mmol m^−2^ s^−1^	mg g^−1^	mg g^−1^	mg g^−1^
0	−AMF	7.9 ± 1.6a	0.7 ± 0.07a	333.3 ± 8.9c	4.0 ± 0.2a	4.7 ± 0.21a	2.6 ± 0.17a	7.3 ± 0.32a
	+AMF	8.5 ± 0.76a	0.8 ± 0.03a	332.7 ± 3.9c	3.9 ± 0.07a	4.8 ± 0.17a	2.8 ± 0.07a	7.6 ± 0.16a
300	−AMF	4.5 ± 0.65c	0.5 ± 0.03b	348.09 ± 6.7b	3.1 ± 0.2b	3.7 ± 0.16b	1.9 ± 0.06b	5.6 ± 0.14c
	+AMF	7.3 ± 0.4ab	0.8 ± 0.06a	326.4 ± 6.9c	4.2 ± 0.2a	4.4 ± 0.27a	2.4 ± 0.1a	6.8 ± 0.32ab
600	−AMF	3.5 ± 0.51d	0.3 ± 0.004c	365.2 ± 5.4a	1.9 ± 0.1c	2.9 ± 0.14c	1.5 ± 0.1c	4.4 ± 0.24d
	+AMF	4.8 ± 0.13c	0.5 ± 0.05b	342.8 ± 3.9b	2.8 ± 0.2b	3.1 ± 0.22c	1.7 ± 0.1bc	4.8 ± 0.28d

Data are the means of three replicates (mean ± SE). Different lowercase letters show significant differences between treatments at *p* < 0.05.

## Data Availability

The data presented in this study are available on request from the corresponding author.

## Supplementary Materials

The following supporting information can be downloaded at: https://www.mdpi.com/article/10.3390/jof8050513/s1, Table S1: Primers of *C. indica* genes used for the qRT-PCR analysis.
