# Supplementary material for: Impact of the Arbuscular Mycorrhizal Fungus Funneliformis mosseae on the Physiological and Defence Responses of Canna indica to Copper Oxide Nanoparticles Stress
Source: J Fungi (Basel). 2022 May 16;8(5):513. doi: 10.3390/jof8050513 (PMC9146287; doi:10.3390/jof8050513)
Supplement: Supplementary file 1 [file jof-08-00513-s001.zip › jof-1711704-supplementary.pdf]

Supplementary Materials: Effects of Arbuscular Mycorrhizal Fungi on *Canna indica* Growth, Antioxidant Enzyme Activities, ROS, and Organic Acid Secretion and Stress-Response Gene Expressions Under Copper Oxide Nanoparticles Stresses

Jie Luo, Qiuxia Yan, Guo Yang and Youbao Wang

Table S1. Primers of *C. indica* genes used for the qRT-PCR analysis

Gene	Description	Gene no.	PCR primiers（5′→3′）
18S rRNA	18S rRNA (guanine-N(7))-methyltransferase	c99859.graph_c0	GACCTACTTGTAGTATAGTTGGGGT<br>TTGCGTGCCTCCGTTTC
Cu-Zn SOD	superoxide dismutase [Cu-Zn] 1	c79685.graph_c0	TGGGCTGGGTCTAATGTGC<br>AGAAAGAGCAGAGGATGGGGAAGAA
GR	probable glutathione S-transferase parA	c100781.graph_c0	GCCTGTTTGCCTACGGAACCAATAT<br>AGCAGCACCAGCCCTTT
POD	peroxidase	c98774.graph_c2	GGGGGAAGAAGCAATTTCATCATCT<br>GCGAAGCAGTCGTGGAA
Nramp2	metal transporter Nramp2 -like	c158116.graph_c0	ACCGAGAGCTTAAGAGTAACGCGCT<br>AGCGCGTTACTCTTAAGCTCTCGGT
Nramp5	metal transporter Nramp5-like	c109375.graph_c1	TGGAAGAAGTTCCTGGCTCACGTTG<br>GCCAAGGACACAAGAAATCCGGGTC
MT2c	metallothionein-like protein 2c	c100495.graph_c0	CGTCTCCACCATATCTCAAGCCACA<br>TCCTGCTGTGGCTTGAGAT
MT2a	metallothionein-like protein 2a	c102948.graph_c1	TCGTGCTCATATCTTAGTCGATCCA<br>GAAGAACGTGTTGGATCGACTAAGA
COPT2	copper transporter 2-like	c103133.graph_c2	ATGACCTTCTTATGGGGCAAGAACT<br>CAAGCATCACGAGGTAGGC
COPT6	copper transporter 6-like	c98923.graph_c0	CCTTCCTCCTCCGCTTCTCCTCAAA<br>TCTTGGGAAGTATCAGGGTTT
MDH1	malate dehydrogenase	c78333.graph_c1	AGTCACTGAGAGTTTGTAGTTCACA<br>CCTTCGCTGTGAACTACAAACT
MDH2	malate dehydrogenase	c99859.graph_c0	GCCTACTATAAATATGAGAGAGAGC<br>GCTCTCTCTCATATTTATAGTAGGC

ALMT1	aluminum-activated malate transporter	c79685.graph_c0	TCCTGTAACCTCAGGTTGACCACCC<br>TTGTGCTCAGGCTGTCTACC
ALMT2	aluminum-activated malate transporter	c100781.graph_c0	GCGTCGACCCACTCCTCCTTGACGT<br>GAGGAAGATAAGGAGGGAGATG
CS1	citrate synthase	c98774.graph_c2	TCACGTGAGCTCTCATCCCCATCTC<br>AGCAACCCTGAGACCAAACT
CS2	citrate synthase	c158116.graph_c0	TCGAACTATCTCCTGTTGGATTGGA<br>CGGGCTTCACGACCAAT
